# Impregnation of Silica Gel with Choline Chloride-MEA as an eco-friendly adsorbent for CO_2_ capture

**DOI:** 10.1038/s41598-024-66334-0

**Published:** 2024-07-02

**Authors:** Maryam Jahanbakhshi, Ahad Ghaemi, Maryam Helmi

**Affiliations:** School of Chemical, Petroleum and Gas Engineering, Iran University of Science and Technology, Tehran, Iran

**Keywords:** Deep eutectic solvent, CO_2_, Adsorption, Choline chloride, Monoethanolamine, Environmental chemistry, Environmental impact, Chemical engineering

## Abstract

Deep eutectic solvents (DES) are a generation of ionic liquids that benefit from low cost, good stability, and environmental-friendly features. In this research, a porous silica gel was impregnated with a eutectic Choline Chloride-Monoethanolamine solvent (ChCl-MEA) to greatly improve its CO_2_ capture performance. In the impregnation, the weight percentages of ChCl-MEA were used in the range of 10–60 wt% at a temperature of 25 °C. The effect of ChCl-MEA loading on the structural properties of the DES-modified silica samples was studied by BET, FTIR, and TGA analyses. Investigation of the CO_2_ adsorption performance at different operational conditions showed that the modified silica gel with 50 wt% ChCl-MEA (Silica-CM50) presents the highest CO_2_ capture capacity of 89.32 mg/g. In the kinetic modeling, the fractional order model with a correlation coefficient of 0.998 resulted in the best fit with the experimental data. In addition, the isotherm data for Silica-CM50 were well-fitted with the Dual site Langmuir isotherm model with a correlation coefficient of 0.999, representing two distinct sites for the adsorption process. Moreover, the thermodynamic parameters including Enthalpy, Entropy, and Gibbs free energy at 25 °C were obtained to be − 2.770, − 0.005 and − 1.162, respectively. The results showed the exothermic, spontaneous and feasibility of the adsorption process.

## Introduction

With increasing levels of air pollutants from fossil fuel combustion and industrial sources and the significance of the greenhouse effect^1^, scientists have predicted that the average global temperature will rise by several degrees^2^. Due to the effects of increasing temperature on the environment and ecosystems of different countries, a lot of research has been done in this field, which has led to the presentation of different technologies for the capture and storage of carbon dioxide^3–5^. The Intergovernmental Panel on the Nobel Prize in Climate Change (IPCC) reports that CO_2_ emissions will be reduced by about 50–80 percent by 2050 as a result of progress in controlling carbon dioxide emissions^6–8^. An example of significant progress in this area is the use of green solvents, which are cheap, non-toxic, and degradable^9^ to solve the problems caused by the use of aqueous alkanolamines (corrosion and high energy consumption)^10–13^. Generally, three techniques were used for CO_2_ capture including pre-combusition, post-combusition, and oxy-fuel^14^. Post-combustion CO_2_ capture, which adsorbs CO_2_ molecules after composition by dry/wet adsorbs, is seen as a promising technique for trapping and storing CO_2_ molecules^15^.

Among traditional organic amines, ionic liquids (ILs) are considered environmentally friendly adsorbents used in different processes like gas separation because of their excellent selectivity and high thermal stability. ILs can absorb CO_2_ effectively due to their easy alkylation in alkaline conditions. A notable IL is the deep eutectic solvent (DES) made up of a hydrogen bond acceptor (HBA) and a hydrogen bond donor (HBD), leading to liquid blends with lower melting points than the individual compounds^16^.

Smith et al.^17^, described DES that are deemed as an attractive alternative to ionic liquids. DESs, unlike ionic liquids, have very low cost, simple preparation methods, and better biodegradability, so are known as green solvents^18^. A DES is synthesized by mixing a hydrogen bond acceptor (HBA) and a hydrogen bond donor (HBD), the resulting solution has a melting point lower than the pure compounds of which the DES is prepared^19,20^. Pressure, temperature, nature, symmetry of the salt, alkyl chain length in HBA and HBD, nature of the HBD and molar ratio affect the solubility of CO_2_ in the DES^21^.

However, due to its high viscosity and the need for special operating conditions (high pressure 5 bar and low temperature), there are limitations to the use of these solvents in carbon dioxide capture^22–24^. One of the suggestions to overcome this problem is to fix DESs on the porous solid materials with large surface area and pore volume^25,26^. Due to the high thermal stability, uniform particle size, ecfriendly, moisture tolerance, and excellent regeneration process, silica gel can be a good choice to solve this problem^27–29^. According to the studies, to enhance adsorption capacity of silica gel, increasing both selectivity and stability of it, silica gel is better to increase the adsorption rate by functionalization of the silica gel with DES^30^.

ChCl-MeA is an abbreviation for choline chloride-methanol adduct, a deep eutectic solvent (DES) created by combining choline chloride and methanol in a specific ratio. DES, a type of ionic liquid, has lower melting points and is considered more environmentally friendly than traditional solvents. When combined with silica gel, ChCl-MeA could form a solid adsorbent for CO_2_ capture. Silica gel offers a porous structure to trap CO_2_ molecules, while ChCl-MeA can improve selectivity and efficiency. This composite material might be utilized in carbon capture and storage technologies to reduce CO_2_ emissions from industrial processes or power plants^31–33^.

Some successful research has been done on these adsorbents to check out their properties and capacity to adsorb carbon dioxide as shown in Tables 1 and 2. Jiao et al. studied CO_2_ adsorption capacity by amine-functionalized mesoporous silica and showed the high CO_2_ adsorption capacity and the ability to overcome toxicity, corrosion, and high energy for desorption of amine solution^34^. Ren et al. investigated that by confinement of IL into PMMA bead, CO_2_ adsorption capacity was improved^35^. Moreover, Zulkurnai et al. measured CO_2_ adsorption capacity by DES-functionalized activated carbon and reported higher CO_2_ adsorption capacity than the non-functionalized activated carbon^36^.

**Table 1:** Solubility of CO_2_ in different DESs at different molar ratios, temperatures and pressures.

Researchers	Year	DES	Molar ratio	T (K)	P (MPa)	qCO2 (mg/g)	References
Serrano et al	2012	ChCl: Urea	1:2	303.15	0.29	12.240	^22^
Leron et al	2013	ChCl: Glycerol	1:2	303.15	0.19	6.370	^23^
Leron et al	2013	ChCl: Ethylene glycol	1:2	303.15	0.24	4.720	^37^
Ma et al	2017	TPAC:EA: AC	1:2:2	298.15	2.00	140.000	^38^
		Benzyltriphenylphosphonium chloride: Glycerol	1:12			0.021	
Ali et al	2014	Methyl triphenyl phosphonium bromide: Ethanol Amine	1:6	298.15	1.00	0.072	^39^
		Tetra Butyl Ammonium Bromide: Ethanol Amine	1:6			0.059

**Table 2:** CO_2_ adsorption capacity at different samples.

Basis adsorbent	Solvent	CO_2_ adsorption capacity (mg/g)	T (°C)	P (atm)	Gas	References
Silica-gel	PEI	44.0	35	1.0	CO_2_	^40^
Silica-gel	PEI	55.0	45	0.1	CO_2_	^41^
Bentonite	PEI	47.0	75	1.0	CO_2_	^42^
Nanoporous silica	ChCl:U	23.0	–	–	CO_2_	^43^
Nanoporous silica	ChCl:Gly	20.1	25	–	CO_2_	^44^
	ChCl:EG	18.8				
Silica-gel	ChCl:U:PEI	51.0	25	1.0	CO_2_	^45^
	ChCl:PEI	43.0				
	PEI	48.0				
PHE	CNT-DES	298.0	–	–	CO_2_	^46^
CRV	CNT-DES	394.0				
Activated carbon	DES ( ChCl and Glycerol)	9. 8	–	–	CO_2_	^36^
		9.5			N_2_	

This study aimed to compare the CO_2_ adsorption capacities of bare Silica gel and Silica_CMx for CO_2_ capture. Initially, our team impregnated silica gel with Choline chloride_MEA in varying ratios to find the best ratio between Silica gel and Choline Chloride-MEA. The distinctive aspect of our study was the impregnation process with Choline chloride_MEA, which enhanced the CO_2_ adsorption properties of the silica gel. This innovative approach differentiates our study from prior research on CO_2_ capture using silica materials. The primary goals of our experiment were to assess the impact of Choline chloride_MEA impregnation on enhancing CO_2_ adsorption capacity and to compare the efficacy of silica_CMx with bare silica gel in CO_2_ capture. The characterization of prepared adsorbents was determined by X-ray diffraction (XRD), Emission Scanning Microscopy (SEM), Fourier transform infrared spectroscopy (FTIR), and Thermogravimetric (TGA) analysis. The kinetic, isotherm, and thermodynamic studies were performed for both adsorbents and their results were compared.

## Experimental methods

### Materials

Choline chloride(ChCl), Monoethanolamine (MEA), Silica gel and Methanol, all with mass fraction purity ≥ 98.0%, were purchased from Merck chemical company. The pure carbon dioxide and nitrogen (purity > 99.0%) were supplied from Hamta Gas Co.

### Preparation of deep eutectic solvent

To prepare a deep eutectic solvent consisting of choline chloride and Monoethanolamine with a molar ratio of 1:8 (ChCl: MEA), the desired amounts of these substances were placed in a closed bath using a closed vial (Fig. 2). To homogenize the solvent, the solution is stirred at a temperature of 60–70 °C for 30 min using a heater to obtain a uniform solution. The resulting solution was left at the ambient temperature for 24 h and after that used to modify the adsorbent.

### Preparation of DES impregnated silica gel

**Figure 1:**
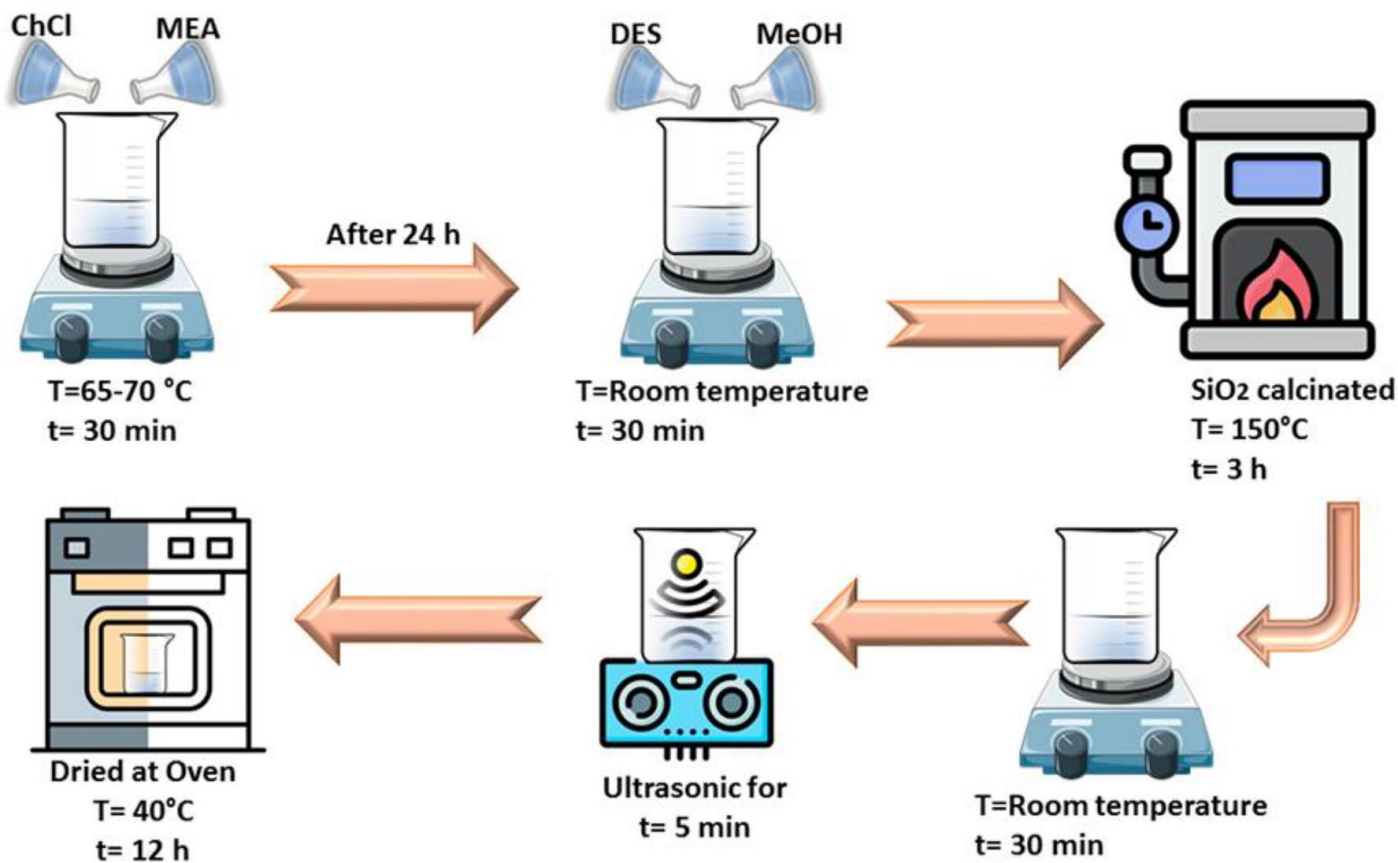
Schematic diagram of adsorbent fabrication.

**Figure 2:**
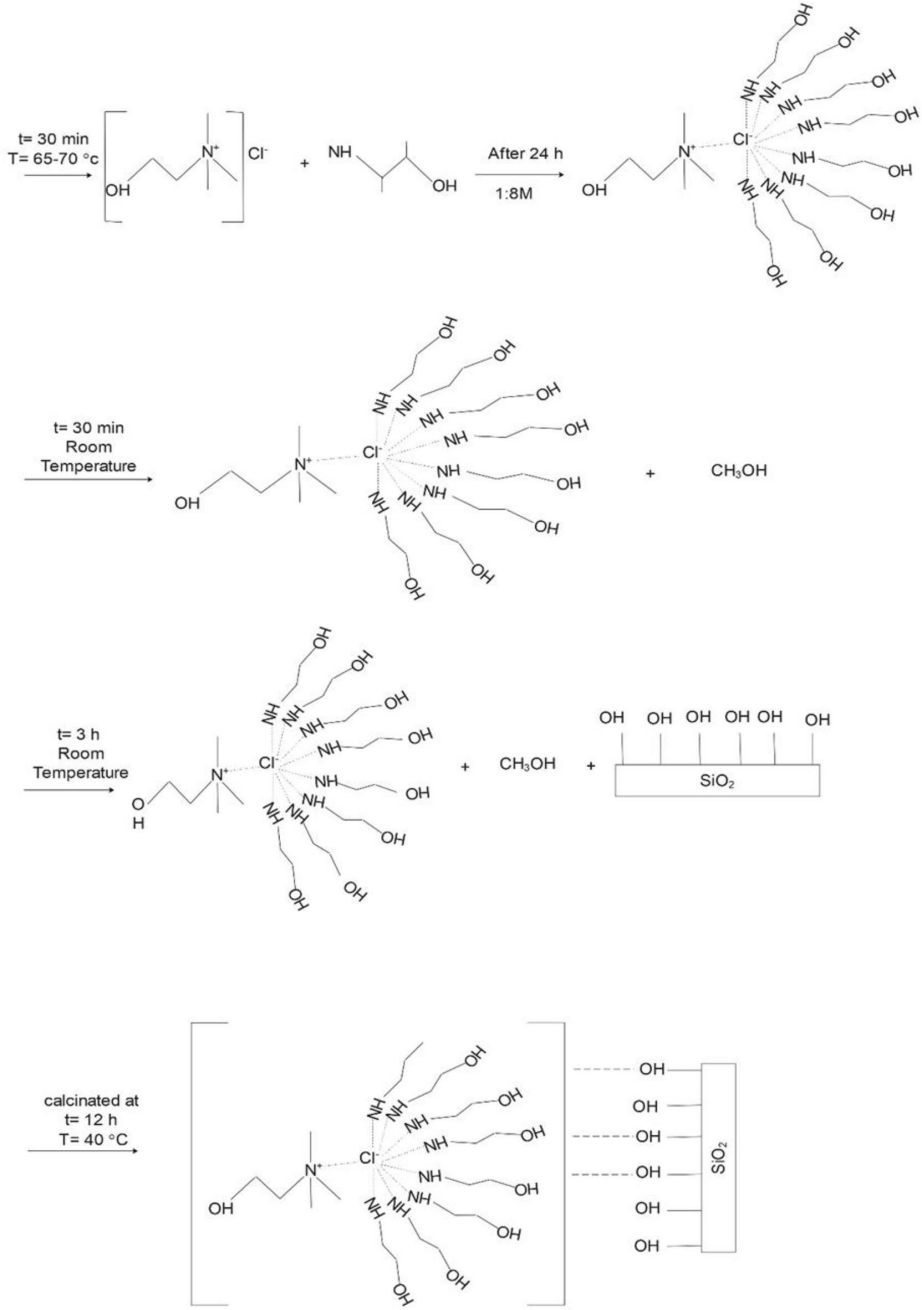
Formulation of the synthesis reaction.

Due to the importance of using porous adsorbents in the CO_2_ capture, as shown in Figs. 1 and 2. In this research silica gel were modified by the impregnation method, with eutectic solvent. First, silica gel was dried at 150 °C for 3 h in a vacuum oven to remove its moisture. After that, different weight percentages of eutectic solvent (10–60 wt%) were stirred with methanol for 30 min at room temperature. After complete dissolution, each solution was poured into a vial with silica gel and placed in ultrasonic for 5 min and then stirred at room temperature for 3 h. The methanol solvent was removed at room temperature and finally, the resulting samples were dried in an oven at 40 °C for 24 h to obtain the desired adsorbent powder. The obtained DES-modified samples were named as Silicon Varbide-Carbon–Metal x (Silica-CMx), where x refers to the weight percent of ChCl-MEA. All procedures and formulation of the synthesis reaction are presented in Fig. 2.

### Characterization methods

The presence of surface functional groups was determined using a Fourier transformed-infrared 8400S spectrometer (Shimadzu Corporation, Japan). The surface area and porosity parameters of the samples were specified using N_2_ physisorption at 77 K using a Micromeritics ASAP 2020 analyzer. Brunauer, Emmet and Teller (BET) method was used to determine the surface area of the adsorbents. Pore volumes and pore size distribution were calculated by the Barret–Joyner–Halenda (BJH) method. In addition, Thermogravimetric analysis (TGA) was used for thermal analysis of the adsorbent. The surface morohology of samples was studied by scanning electron microscopy (SEM) analysis.

### CO_2_ adsorption experiments and setup

**Figure 3:**
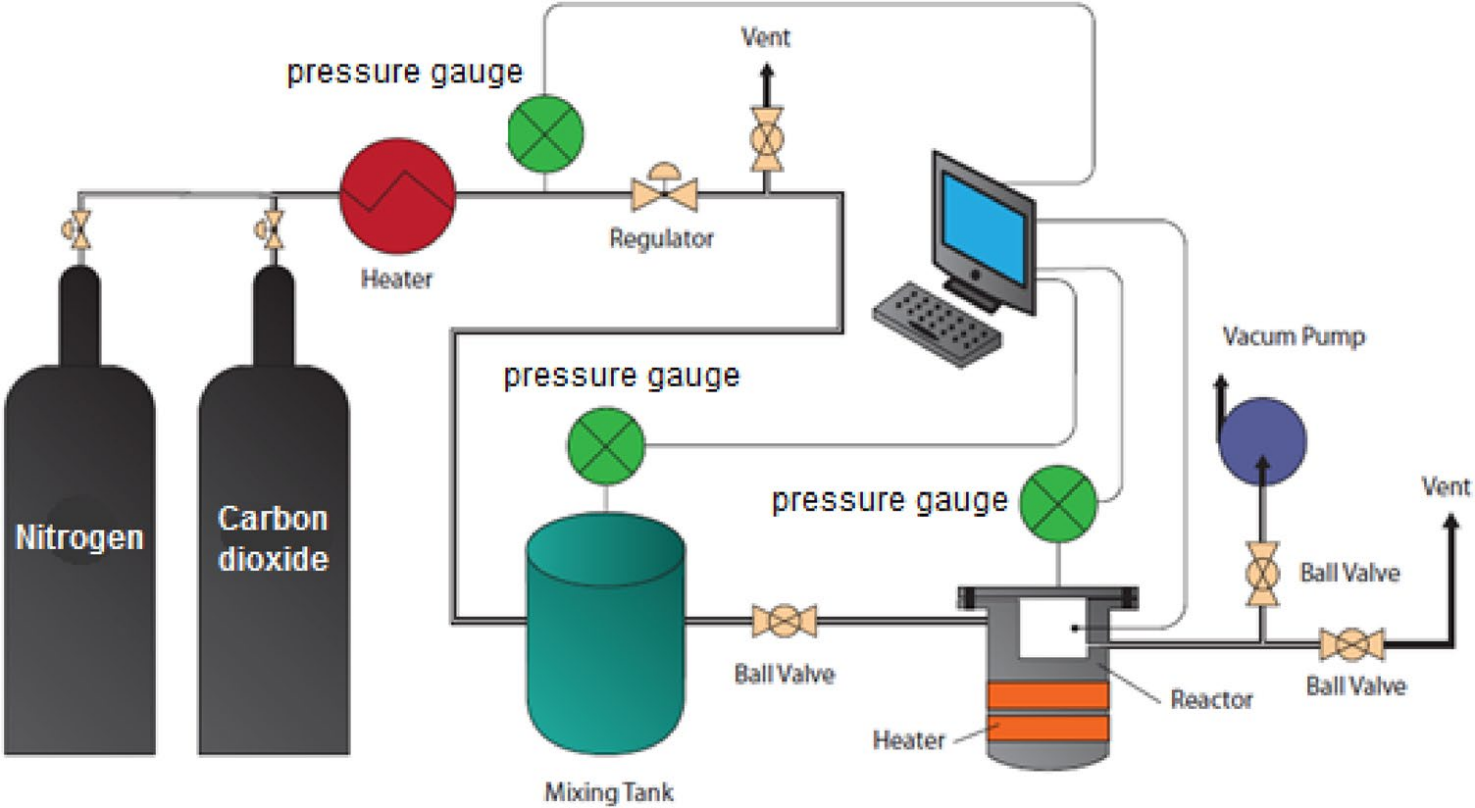
Setup of CO_2_ adsorption.

Carbon dioxide adsorption was performed on bare silica gel and modified silica gel at room temperature using a system consisting of a stainless-steel batch reactor, which includes inlets and outlets for gas to pass through the adsorbent surface^13^. As shown in Fig. 3, this experimental set-up was equipped with a pressure regulator and a thermocouple to adjust the pressure and desired temperature, respectively. The adsorbents, which were prepared in different weight percentages, were placed inside the reactor using a mesh-shaped chamber. At this stage, the temperature of the device is adjusted and the reactor door is completely closed to isolate the system so that the thermal balance between the adsorbent and the system can be achieved.

As the gas enters the reactor, the adsorption operation begins. It must be ensured that the reactor outlet valve is closed at this stage. At the start of the operation, information about the pressure and temperature of the reactor can be seen by the system monitor. By checking the observed pressure drop when this value is almost constant, it can be considered as the equilibrium time and the end of the adsorption operation. All required data including temperature, pressure and adsorption time during the adsorption process were recorded by the system.

### CO_2_ adsorption calculations

The moles of CO_2_ adsorbed into the DES-based sorbent ($n_{{CO_{2} }}^{L}$) was calculated as follows:

1 $$ P_{{CO_{2} }} = P_{total} - P_{solution}^{sat} $$

2 $$ n_{{CO_{2} }}^{G} = \frac{{V_{G} P_{{CO_{2} }} }}{RTZ} $$

3 $$ \left. {n_{{CO_{2} }}^{L} } \right|_{t} = \left. {n_{{CO_{2} }}^{G} } \right|_{initial} - \left. {n_{{CO_{2} }}^{G} } \right|_{t} $$

4 $$ Z^{3} - Z^{2} + (\frac{{aP - b^{2} P^{2} }}{{R^{2} T^{2} }} - \frac{bP}{{RT}})Z = \frac{{abP^{2} }}{{R^{3} T^{3} }} $$

5 $$ a = 0.4274\frac{{R^{2} T_{c}^{2} }}{{P_{c} }}[1 + m(1 - \sqrt {T_{r} } )]^{2} $$

6 $$ b = 0.0866\frac{{RT_{c} }}{{P_{c} }} $$

7 $$ m = 0.48 + 1.574\omega - 0.176\omega^{2} $$

where *P*_total_ is the total inner pressure of the reactor,$P_{solution}^{sat}$ is the vapor pressure of the aqueous alkanolamine solution at the given temperature, R is the gas constant, T_c_ is the critical temperature, T_r_ is the reduced temperature, P_c_ is the critical pressure and $\omega$ is the acentric factor, Z is the compressibility factor and calculated using Soave–Redlich–Kwong (SRK) equation of the state. Superscripts G and L refer to the gas and liquid phases, respectively^47^.

## Result and discussion

### Characterization of DES-modified silica gel

**Figure 4:**
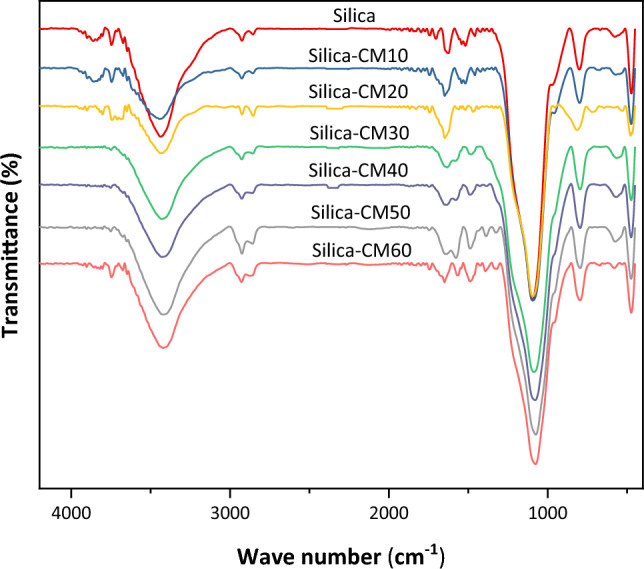
FTIR spectra for pure silica gel and DES-modified silica with six different weight percent.

FTIR spectra of the experimental samples of silica gel modified by eutectic solvent with six different weight percentages and a sample of bare silica gel were examined. An infrared spectrometer (Shimadzu Corporation, Japan) was used for spectral analysis. The FTIR spectra of the samples can be seen in Fig. 4. Peaks at 800.74 cm^−1^ and 1098.11 cm^−1^ corresponded to Si–O and Si–O–Si of silica gel, respectively^48^. The peak at 3438.51 cm^−1^ was assigned to the N–H bonding from choline chloride^49^. The peak at 1100 cm^−1^, which shifted to 1070 cm^−1^, can be linked to the C–N bonding^48^. Haider et al. reported that due to the formation of hydrogen bonds in the DES, peaks have the same wavenumbers, therefore, through the formation of different types of hydrogen bonds there is some physical interaction^50^. The presence of amine in the silica gel can be detected with the presence of the peak at 2900 cm^−1^, which i intensified as the DES load is increased.

**Figure 5:**
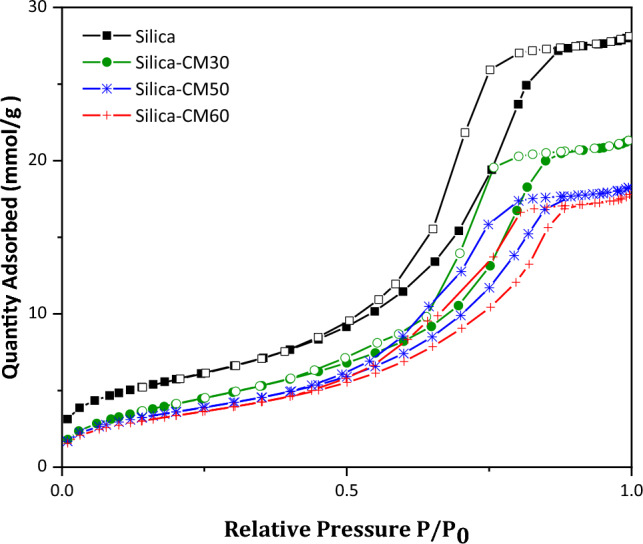
Nitrogen adsorption and desorption isotherm for the bare silica and DES-modified silica.

Nitrogen adsorption and desorption analysis were used to show the surface properties and porosity of silica gel modified by eutectic solvent and pure silica gel. Samples were placed in a vacuum oven for resuscitation before analysis. In this analysis, the samples of pure silica gel and modified silica gel with 30, 50 and 60% have been used. Also, the analysis has been done using a Micromeritics ASAP 2020. To explain the isotherms, Brunauer, Emmett and Teller (BET) classification was provided^51,52^. According to Fig. 5, the adsorption and desorption isotherms for all the samples belong to type IV, which according to IOPAC classification indicates the mesoporous of the adsorbent samples^53^. The results of the sample analysis are given in Fig. 5. Nitrogen adsorption and desorption isotherm for the bare silica and DES-modified silica.

Table 3. According to the results, it can be seen that the specific surface area of the adsorbent is reduced and the pore size is increased with increasing the weight percentage of the eutectic solvent, which may be due to the pore blocking, pore filling, and thickening of ChCl-MEA layer onto the silica gel^25,52,54^.

**Table 3:** BET Analysis Results for the bare silica and DES-modified silica samples.

Samples	Specific surface area (m^2^ g^-1^)	Pore volume (m^3^ g^-1^)	Average pore diameter (nm)
Silica	466.91	0.964	8.260
Silica-CM30	350.75	0.729	8.315
Silica-CM50	299.43	0.624	8.338
Silica-CM60	279.93	0.605	8.640

**Figure 6:**
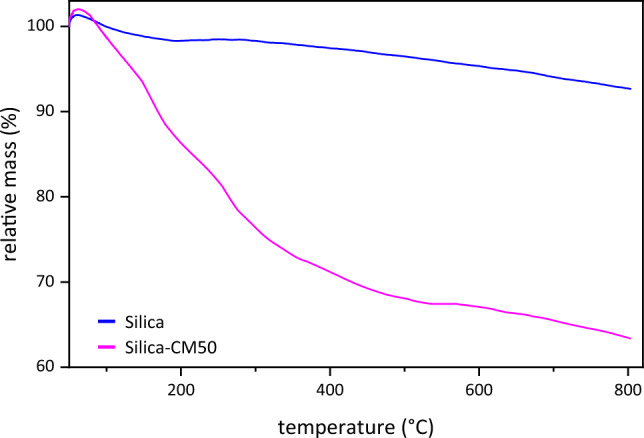
TGA Analysis results for the bare silica and Silica-CM_50_.

Figure 6 illustrates temperature decomposition of bare silica and Silica-CM50. According to the Fig. 6, bare Silica did not display any weight loss at high temperature. That is because Silica nano-particles had high thermal stability^55^. However, by increasing temperature to 200 °C, moisture of both sample disappeared. In the TGA analysis of Silica_CM50, weight loss happens in two stages. Initially, as the temperature rises to 300 °C, the sample's moisture evaporates. Subsequently, as the temperature climbs to 400 °C, the impregnated Choline chloride_MEA decomposes and gets removed from the silica gel matrix^56^. The weight loss seen in the TGA analysis stems from the thermal breakdown of the impregnated material when exposed to higher temperatures. This decomposition results in the liberation of gases and volatile substances, leading to a reduction in the sample’s 35% overall weight^38^.

**Figure 7:**
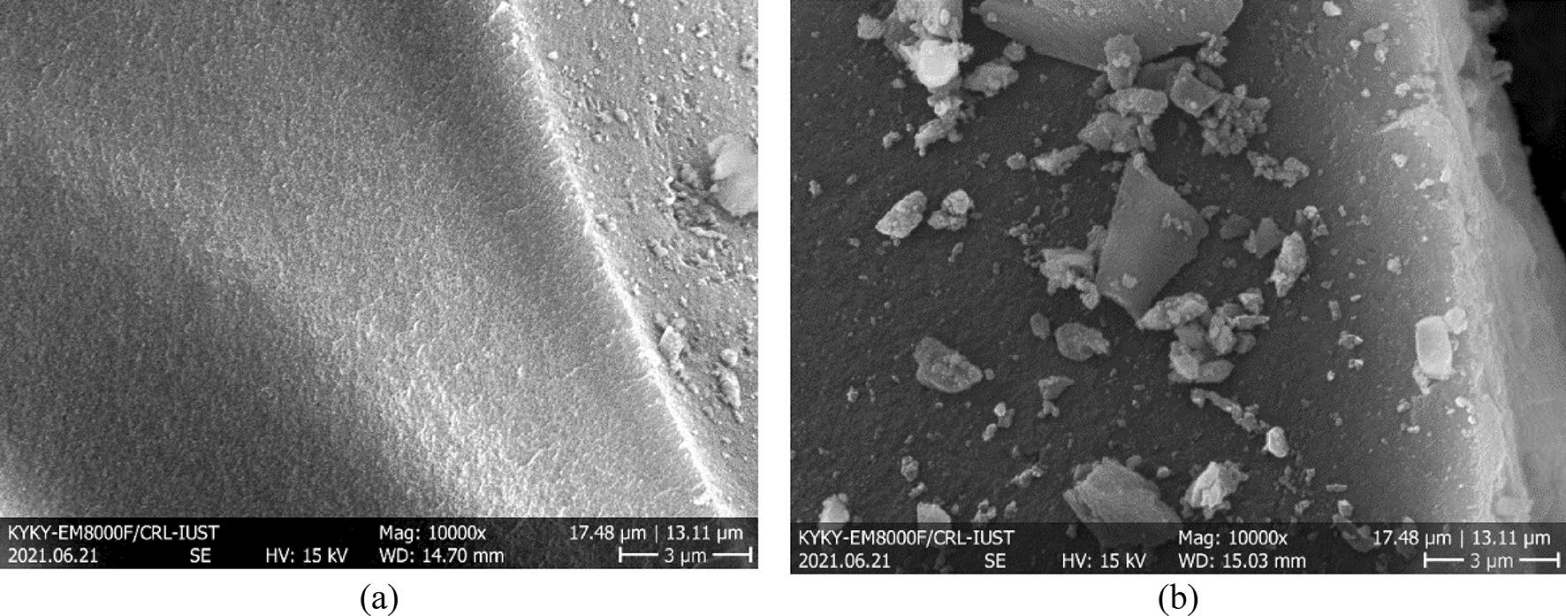
SEM micrograph for (**a**) bare Silica, (**b**)Silica-CM50 sample.

Figure 7 shows the Silica-CM50 sample before exposure with CO_2_ and bare Silica morphology. There were unclear changes in the sample structure after the modification with Choline Chloride-Monoethanolamine. The Silica surface was covered with ChCl: MEA, therefore, the presence of the pore on the surface of silica could not be clearly seen.

### CO_2_ capture capacity

**Figure 8:**
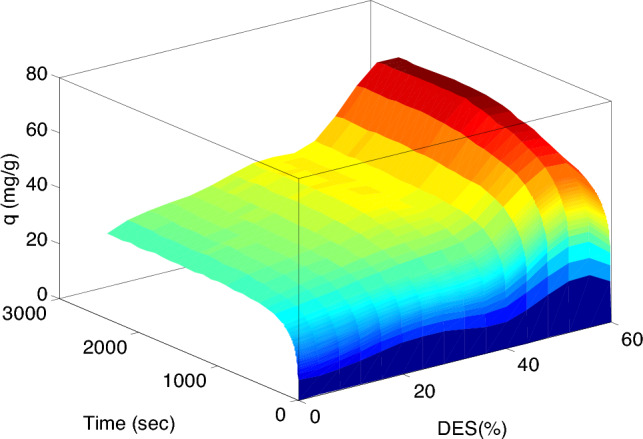
Experimental equilibrium data for CO_2_ adsorption at bare silica and Silica-CM_50_.

To compare the CO_2_ adsorption capacity of the bare silica and Silica-CM_x_ samples, the adsorption experiments were performed at 25 °C and an initial pressure of 6 bar. As can be seen in Fig. 8, the CO_2_ adsorption capacity increases with increasing DES loading on silica up to 50 wt% and then the CO_2_ uptake decreases for the silica-CM60 sample. Modification of silica gel with ChCl-MEA enhances the CO_2_ capacity from 31.02 mg/g to 35.77, 38.72, 43.69, 45.54, 65.78, and 56.628 mg/g for 10 to 60 wt% DES loading, respectively. According to Malherbe et al.^57^, Bare silica mainly adsorbs CO_2_ through physical interactions such as van der Waals forces and electrostatic interactions. On the other hand, DES-modified silica can enhance CO_2_ adsorption due to the functional groups in the DES that can chemically interact with CO_2_ molecules, leading to stronger adsorption. Additionally, specific functional groups in the DES, like amino groups or hydroxyl groups, can assist in the chemisorption of CO_2_ through mechanisms like hydrogen bonding or acid–base interactions. Understanding these interactions at a molecular level is crucial for elucidating the CO_2_ adsorption process of DES-modified silica and improving its performance for practical applications^58^. Hence, despite a significant drop in surface area with DES loading, the higher CO_2_ uptake of Silica-CM_x_ samples could be attributed to the strong interactions between CO_2_ and Silica-CM_50_.

**Figure 9:**
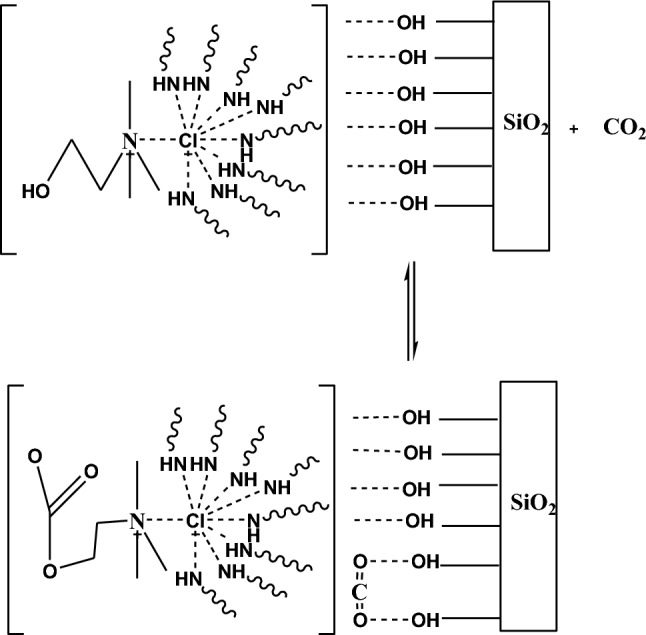
Mechanism of CO_2_ adsorption using Silica-CM50.

Decrease in CO_2_ adsorption capacity can be attributed to several factors. Initially, bare silica primarily adsorbs CO_2_ through physical adsorption, where CO_2_ molecules are attracted to the surface of the silica via weak van der Waals forces. Conversely, DES-modified silica can demonstrate improved CO_2_ adsorption performance due to the presence of DES molecules, which can interact with CO_2_ through specific chemical interactions like hydrogen bonding or Lewis acid–base interactions. Moreover, the presence of DES molecules on the silica surface can enhance CO_2_ adsorption by offering additional adsorption sites and facilitating specific interactions with CO_2_ molecules. Nevertheless, at high DES loadings, these advantages may be offset by factors such as pore blockage or competitive adsorption, resulting in a reduction in CO_2_ uptake^59,60^. Figure 9 shows mechanism for CO_2_ capture by Silica-CM_50._

### Adsorption kinetics

Three widely applied kinetic models, including pseudo first, second, and fractional order models were applied to study the kinetic performance of the Silica-CM_x_ samples for CO_2_ capture. The kinetic models and their integrated forms after applying the boundary condition (q_t_ = 0 at t = 0 and q_t_ = q_e_ at t = ∞) are written as follows:

Pseudo first order^61^:

8 $$ \frac{{\partial q_{t} }}{\partial t} = k_{1} (q_{e} - q_{t} ) $$

9 $$ q_{t} = q_{e} (1 - e^{{ - k_{1} t}} ) $$

Pseudo second order^62^:

10 $$ \frac{{\partial q_{t} }}{\partial t} = k_{2} (q_{e} - q_{t} )^{2} $$

11 $$ q_{t} = \frac{{q_{e}^{2} k_{2} t}}{{1 + q_{e} k_{2} t}} $$

**Figure 10:**
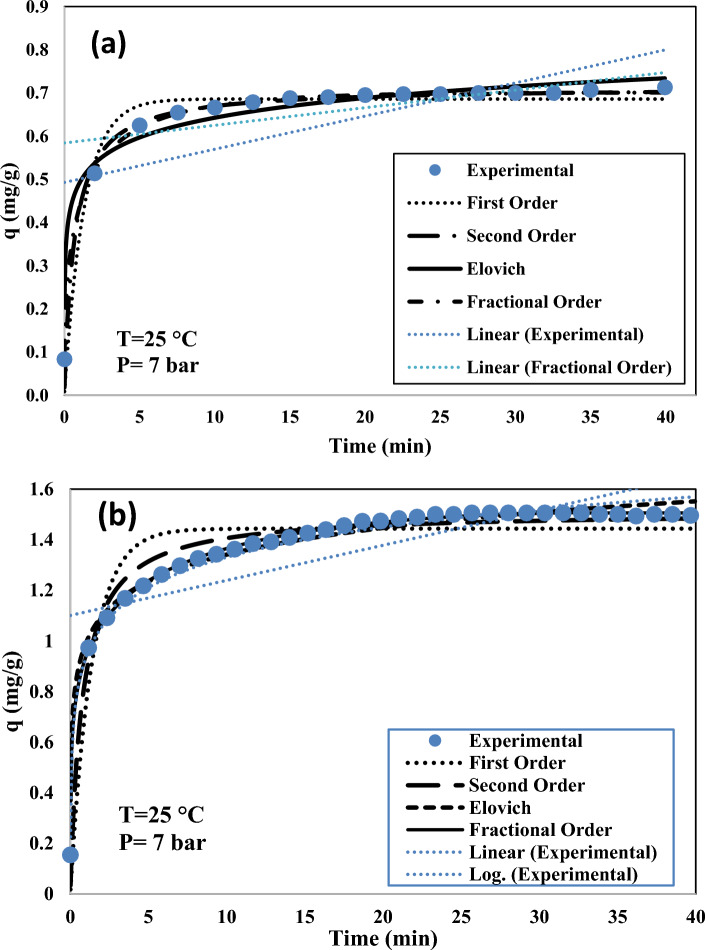
Comparison of pseudo first, second, Elovich and fractional order kinetic models for (**a**) silica gel, (**b**) Silica-CM50.

Fractional order^63^:

12 $$ \frac{{\partial q_{t} }}{\partial t} = k_{n} t^{m - 1} (q_{e} - q_{t} )^{n} $$

13 $$ q_{t} = q_{e} - \frac{1}{{\left( {\frac{{(n - 1)k_{n} t^{m} }}{m} + \frac{1}{{q_{e}^{n - 1} }}} \right)^{1/(n - 1)} }} $$

where q_t_ and q_e_ are the adsorption capacity at the time t and at equilibrium, respectively, k_1_ and k_2_ are the pseudo first order and pseudo second order adsorption rate constants, respectively, and k_n_, m, and n are the constants for the fractional order kinetic model. To obtain kinetic information about CO_2_ adsorption onto Silica-CM_x_ samples pervious mentioned kinetic models were fitted to the experimental data. For instance, the curves generated by kinetic models as well as the experimental data for bare silica and silica-CM_50_ are depicted in Fig. 10a,b.

To enhance the adsorption process, kinetic modeling was employed to specify residence time and adsorption rate. The kinetic models assist to measure adsorption mechanism and adsorption rate as well. All experimental runs were performed at 25 °C and 7 bar^64,65^. The corresponding kinetic parameters, coefficient of determination (R^2^) for regressions and error are summarized in Table 4. The pseudo first-order model is often employed to estimate the kinetic of CO_2_ physisorption on solid sorbents^66,67^. This model does not properly fit with the experimental data at the initial and final stage of adsorption, especially at higher ChCl-MEA loadings. In contrast, the pseudo-second-order model presented a better agreement with the experimental data. Other previous studies have also reported some limitations for the pseudo-first and second-order kinetic models to predict CO_2_ adsorption on amine-modified silica^10,63^. The fractional order model, being put forward by Heydari-Gorji and Sayari^63^ to describe CO_2_ capture on polyethylenimine-impregnated mesoporous silica, offers the best description for the CO_2_ adsorption behavior onto Silica-CM_x_ samples over the entire adsorption range. The parameter m of this model refers to diffusion resistance, and n demonstrates the effect of the driving force (number of unoccupied sites)^63,68^. In general, m decreases with increasing ChCl-MEA loading because the pores blocked by excess deep eutectic solvent lead to slower CO_2_ diffusion. In addition, small values of the parameter n indicate that the adsorption rate is less dependent on the driving force at higher ChCl-MEA loading^69^. In the case of CO_2_ adsorption by Silica_CM_50_, both bare silica and deep eutectic solvent (DES) modified silica may fit the fractional order model better for several reasons: The adsorption of CO_2_ on silica surfaces can involve multiple, simultaneous reaction pathways that are not adequately described by simple integer-order kinetics. The fractional order model allows for the representation of these complex pathways more accurately. Both bare and DES-modified silica have heterogeneous surfaces with a variety of active sites. The fractional order model can account for the different reactivities of these sites, which is not possible with integer-order models. The process of CO_2_ adsorption by silica may be limited by diffusion, especially in the pores of the silica matrix. Fractional order kinetics can incorporate the effects of anomalous diffusion, which is more representative of the actual process. The interaction between CO_2_ and the silica surface might involve the formation of intermediate complexes. The kinetics of these complex formations and decompositions can be better described by fractional orders due to their non-integer stoichiometry. The energy barrier for CO_2_ adsorption varies across the surface of the silica. The fractional-order model can accommodate the distribution of these energy barriers better than integer-order models. These reasons contribute to the better fit of the fractional order model for describing the kinetics of CO_2_ adsorption by both bare silica and DES modified silica. It's important to note that the specific reasons can vary based on the characteristics of the silica used and the conditions of the adsorption process^70^.

**Table 4:** Results of CO_2_ adsorption kinetic models from experimental data of bare silica gel and DES-modified silica gel with different ratios.

Kinetic model	Parameters	Units	Model q_t_	Experimental q_t_					
				10	20	30	40	50	60
Pseudo first order	q_t_	mg/g	30.184	34.45	37.44	42.15	44	63.54	55
	k_1_	1/min	0.758	0.673	0.767	0.543	0.871	0.724	0.905
	R^2^	–	0.943	0.923	0.883	0.886	0.897	0.835	0.851
Pseudo second order	q_t_	mg/g	31.42	36.04	39	44.4	45.67	66.53	56.41
	k_2_	g/mg min	2.104	1.57	1.682	1.02	1.641	0.879	1.327
	R^2^		0.988	0.982	0.963	0.955	0.974	0.938	0.954
Elovich	α	–	25,608	7906	15,948	2557	31,254	4471	20,644
	β	–	0.066	0.083	0.084	0.111	0.092	0.152	0.115
	R^2^	–	0.93065	0.964	0.9711	0.9741	0.9589	0.9843	0.9776
Fractional order	q_t_	mg/g	30.844	35.95	38.72	43.6	45.76	66	56.76
	k_n_	g^n-1^/mmol^n-1^ min^m^	0.367	0.37	0.231	0.191	0.385	0.22	0.273
	n	–	0.832	0.946	0.601	0.438	0.914	0.397	0.647
	m	–	0.437	0.424	0.293	0.281	0.389	0.233	0.284
	R^2^		0.998	0.998	0.998	0.996	0.997	0.995	0.997

### Adsorption isotherms

**Figure 11:**
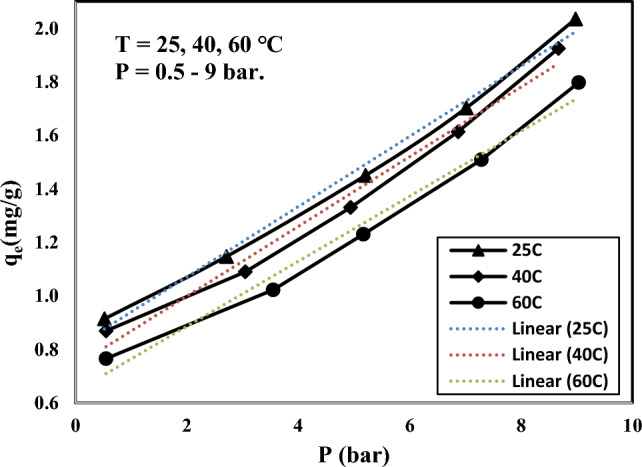
Isotherm data of Silica-CM50 at 25, 40, and 60 °C and in pressure of 7 bar.

The adsorption isotherms can be used to describe the nature of the CO_2_-adsorbent interactions. With that end in view, the experimental equilibrium data were fitted according to the most common isotherm models: Langmuir, Dual site Langmuir, and Freundlich models. Langmuir, the simplest theoretical model, describes a monolayer physisorption or chemisorption over a homogenous surface as follow^71^:

14 $$ q_{e} = q_{m} \frac{bP}{{1 + bP}} $$

where b and q_m_ are Langmuir parameters representing the affinity constant (1/bar) and the maximum monolayer uptake capacity (mg/g). P is the equilibrium CO_2_ partial pressure (bar) and q_e_ refers to the amount of gas adsorbed at this pressure (mg/g). Dual site Langmuir predicts the adsorption over a heterogeneous surface, assuming two distinct adsorption sites would be available to gas molecules:

15 $$ q_{e} = q_{m,1} \frac{{b_{1} P}}{{1 + b_{1} P}} + q_{m,2} \frac{{b_{2} P}}{{1 + b_{2} P}} $$

where the subscripts refer to sites 1 and 2. Freundlich isotherm is the earliest empirical model describing the adsorption process. Freundlich equation can be applied to study the multilayer adsorption of gas molecules on heterogeneous surfaces^72^:

16 $$ q_{e} = k_{f} P^{1/n} $$

where k is the Freundlich constant and n is an indicator of the surface heterogeneity. The accuracy of each model was assessed by coefficient of determination (R^2^) and an average relative error (ARE):

17 $$ ARE (\% ) = \frac{100}{n}\sum\limits_{i = 1}^{n} {\frac{{\left| {q^{\exp } - q^{\bmod } } \right|_{i} }}{{q_{i}^{\exp } }}} $$

where q^exp^ and q^mod^ denote the experimental and model predicted values of adsorption uptakes, respectively, n is the number of experimental data. To find out the temperature influence on the CO_2_ uptake capacity, the equilibrium isotherm data were measured for the Silica-CM_50_ sample, which exhibited substantially higher CO_2_ capacity value among all studied samples. Figure 13 shows the adsorption isotherms of CO_2_ on Silica-CM_50_ at 25, 40 and 60 °C and 7 bar; the fitting of isotherm data with Dual site Langmuir model is illustrated as well. In the studied range of pressure, the maximum CO_2_ capture is 89.32 mg/g, obtained at 25 °C. As can be seen from Fig. 11, at higher temperatures, the CO_2_ adsorption capacity decreases, indicating the exothermic nature of the adsorption process. This could be because with rising temperature, the interaction between CO_2_ molecules and sorbent surface becomes weak and the desorption process is initialized.

To figure out the surface characteristics of Silica-CM_50_, Langmuir, Dual site Langmuir, and Freundlich isotherm models have been fitted to the obtained equilibrium data and the corresponding parameters along with the R^2^ and error values are listed in Table 5. Langmuir model showed a significant deviation from the experimental data which could be because this model assumes a monolayer CO_2_ uptake and does not consider the surface heterogeneity. Freundlich isotherm follows the experimental data more accurately. However, the higher R^2^ and lower error values confirm that the dual-site Langmuir model fits best with the isotherm data. This reflects the heterogeneous nature of the surface which is supported by values greater than one obtained for the n parameter in Freundlich isotherms. Also, the affinity constant b indicates a reducing trend with temperature increasing, verifying the exothermic nature of the CO_2_ uptake process^73,74^.

**Table 5:** Results of CO_2_ adsorption isotherm models from experimental data of Silica-CM50.

Isotherm model	Parameters	Units	Temperature		
			25 °C	40 °C	60 °C
Langmuir	q_m_	mg/g	83.6	79.2	74.8
	b	1/bar	1.13	1.09	0.96
	R^2^	–	0.9665	0.9650	0.9645
	ARE	%	11.33	15.41	15.72
Freundlich	q_m_	mg/g	40.48	37.4	33.44
	n	–	3.07	2.96	2.81
	R^2^	–	0.9881	0.9869	0.9868
	ARE	%	5.60	10.10	10.26
Dual site Langmuir	q_m,1_	mg/g	46.76	44.29	40.43
	b_1_	1/bar	0.0029	0.0030	0.0031
	q_m,2_	mg/g	0.81	0.74	0.65
	b_2_	1/bar	161.90	181.07	178.35
	R^2^	–	0.9989	0.9984	0.9982
	ARE	%	2.70	3.62	3.62

### Adsorption thermodynamic

**Figure 12:**
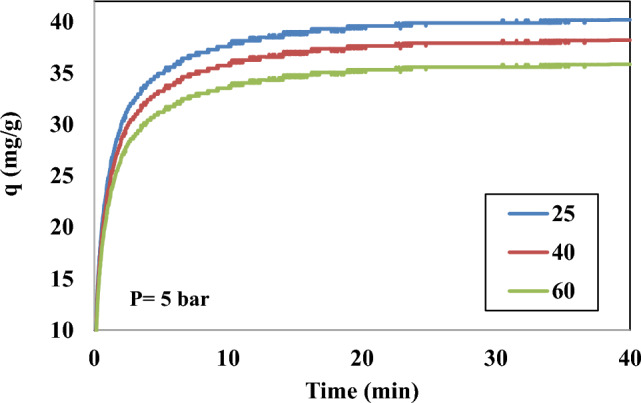
Effect of temperature on uptake of CO_2_ on Silica-CM50 at different temperatures at 5 bar.

To find out the effects of adsorption temperature on DES-modified adsorbent, CO_2_ adsorption curves of Silica-CM50 at various adsorption temperatures are tested, as appeared in Fig. 12. It can be seen that by raising the temperature from 298 to 343 K, a decrease in the uptake ability of CO_2_ can be observed. The thermodynamic parameters are obtained from the adsorption experiments at 298, 313, 328 and 343 K and 5 bar. Thermodynamic parameters, including ΔG, ΔS and ΔH, are used to evaluate the thermodynamic feasibility of the process between gas molecules and Silica-CM50 and to confirm the nature of the uptake process. The laws of thermodynamics shared with the experimentally attained adsorption data obtained from the Langmuir isotherm can be used to evaluate the thermodynamic limitations according to Eqs. (18) and (19):

18 $$ \Delta G = - RT\ln K_{d} $$

19 $$ \Delta G = \Delta H - T\Delta S $$

**Figure 13:**
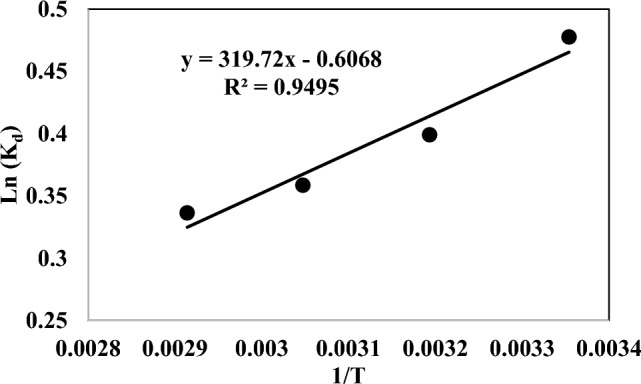
Adsorption isotherm of CO2 on Silica-CM50 at differentj temperatures.

The ΔS and ΔH were determined from the intercept and slope of the linear variation of ln K_d_ against 1/T as shown in Fig. 13, with Eq. (20):

20 $$ Ln \, K_{d} \, = \frac{\Delta S}{R} - \frac{\Delta H}{{RT}} $$

ΔG is used to understand the spontaneity of the adsorption process, which is also a vital factor. The negative ΔG values were acquired for CO_2_ at the three above-mentioned temperatures, reflecting the gas uptake was thermodynamically spontaneous and favourable at low temperatures. The positive or negative ΔS values lead to an increase or a decrease in the randomness at the gas–solid interface during the adsorption process, respectively. The negative ΔS value (0.00541 kj/mol K) demonstrates the decrease in entropy during the adsorption process, which indicates the high order of adsorbent molecules in the adsorption process.

ΔH is a critical parameter to examine whether the adsorption process is exothermic or endothermic according to its negative or positive value. The negative ΔH value (− 2.774 kJ/mol) indicated the exothermic nature of CO_2_ uptake on this synthesized sample, which was the explanation for the reduction in uptake amount at higher temperatures. These results can be attributed to the higher temperatures, the interaction between the adsorbent surface and gas molecules becomes weak and the results in the initialization of the desorption process^68^. The summary of the calculated thermodynamic parameters is listed in Table 6.

**Table 6:** Thermodynamic parameters for CO_2_ adsorption on Silica-CM50.

ΔG (kj/mol)				ΔS (kj/mol K)	ΔH (kj/mol)	− ΔH/R	ΔS/R	R (kJ/ mol K)
55 °C	45 °C	35 °C	25 °C	− 0.0054	− 2.7747	− 319.72	− 0.6505	0.00831
− 0.919	− 1.000	− 1.081	− 1.162					

### Desorption analysis

**Figure 14:**
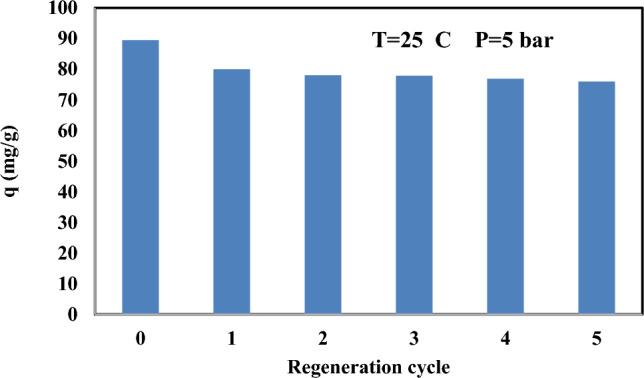
CO_2_ adsorption capacity of bare silica and DES-modified silica after 5 adsorption and regeneration step.

Adsorbent regeneration and activation are the most important issues in the design of adsorbents for practicable applications^75^. The regeneration studies was performed at 25 °C and 9 bar. The feasibility of regeneration was determined by desorption studies for the adsorbent using nitrogen gas. Figure 14 shows the adsorption capacity (q) diagram after 5 steps of CO_2_ adsorption and the nitrogen gas was used to reduce the adsorbent with each adsorption step. The decrease in silica_CM50's ability to regenerate for CO_2_ capture could be due to several reasons. The silica may deteriorate or disintegrate from frequent exposure to high temperatures during regeneration, leading to a decrease in surface area and pore volume, which affects CO_2_ adsorption. Deactivation of active sites by CO_2_ molecules and performance decline caused by material degradation from repeated adsorption-regeneration cycles may also play a role in reducing efficiency over time.

## Conclusion

The primary objective of this investigation is to analyze the CO_2_ adsorption capabilities of untreated silica gel and silica_CMx with varying ratios for CO_2_ capture. Initially, silica gel was impregnated with Choline chloride_MEA in various proportions in order to accomplish this goal. Our research team observed through experimentation that silica_CM50 demonstrated a superior CO_2_ adsorption capacity in comparison to untreated silica gel. The distinguishing feature of our study is the utilization of the impregnation technique with Choline chloride_MEA, which facilitated the enhancement of the CO_2_ adsorption characteristics of the silica gel. This novel methodology differentiates our study from prior investigations in the realm of CO_2_ capture utilizing silica-based materials. Also, by examining the structural properties of the adsorbent, it was founded that the confinement of DES reduced the BET surface area. The presence of surface functional groups was determined using Fourier transformed-infrared 8400S spectrometer and Thermogravimetric analysis was performed for samples. In addition, experiments were performed to investigate isothermal and kinetic models of the adsorption process. It was observed that the isotherm model is consistent with the dual Langmuir isotherm model, which refers to heterogeneous adsorption. The study of kinetic models showed that the fractional order model had the best fit with the adsorption data. The thermodynamic parameters indicated the exothermic nature of CO_2_ uptake on this synthesized sample which was the explanation for the reduction in uptake amount at higher temperature. Silica-CM50 may not be the top performer in CO_2_ adsorption compared to other materials, but it has notable advantages as a CO_2_ adsorbent. These include a high adsorption capacity of 89.32 mg/g at 25 °C, selectivity for CO_2_, stability under various conditions, ease of regeneration for multiple cycles of CO_2_ capture, and the ability to be produced in large quantities at a reasonable cost. Evaluating its potential for cost-effective CO_2_ capture should consider these strengths. Further research may reveal more benefits and optimization strategies for Silica-CM50 in CO_2_ capture applications.

## Data Availability

The data used and analyzed during the current work is available from the corresponding author upon reasonable request.
